# An optimization procedure of shape and position of the aerodynamic device at the rear side of a semi-trailer truck model

**DOI:** 10.1016/j.heliyon.2024.e41411

**Published:** 2024-12-21

**Authors:** Stjepan Galamboš, Nebojša Nikolić, Goran Vorotović, Boris Stojić, Jovan Dorić, Dalibor Feher

**Affiliations:** a Faculty of Technical Sciences, University of Novi Sad, Novi Sad, Serbia; b Faculty of Mechanical Engineering, University of Belgrade, Belgrade, Serbia

**Keywords:** Drag force, Semi-trailer truck, Design of experiments, Airfoil, Wind tunnel testing

## Abstract

The paper outlines the development and optimization of an aerodynamic device for a semi-trailer truck model to reduce aerodynamic drag force. The optimization procedure involves the selection of a basic aerodynamic device shape, using airfoil profiles, and refining its shape and position through established optimization techniques like Full Factorial Design and Response Surface Method within the Design of Experiments framework. The test subject is a 1:10 scale model of the semi-trailer truck. To validate the results obtained from the Computational Fluid Dynamics (CFD) simulations, experimental measurements of aerodynamic drag forces were performed in a wind tunnel. This combination of simulation and experimental testing ensures the reliability and effectiveness of the optimization process for improving vehicle aerodynamics.

## 1 Introduction

Vehicle aerodynamics represents an important scientific discipline that has emerged and developed parallel with vehicle evolution. Its significance became particularly pronounced in the latter half of the twentieth century as awareness of fuel efficiency and the necessity to enhance transport effectiveness increased. The external shape of vehicles gained importance, and aerodynamics became an indispensable component of vehicle design modeling.

A semi-trailer truck is composed of a combination of vehicles, including a truck tractor and the semi-trailer it hauls. These vehicles are generally over 16 m in length, standing 4 m tall and 2.6 m wide. Their box-like shape makes them aerodynamically unfavorable as they move at speeds around 100 km/h. Given the considerable distances covered during typical operation, even minimal aerodynamic improvements aimed at reducing aerodynamic drag force can result in substantial fuel savings.

Hirz and Stadler in their paper [1] emphasize the importance of optimizing aerodynamic devices to save fuel for heavy trucks. They also draw attention to the significance of aerodynamic drag in the equation of forces and the total resistances of vehicles in motion. The aerodynamic impact of vehicle length on the road and the analysis of transport efficiency through Computational Fluid Dynamics (CFD) simulations are demonstrated by Martini et al. in paper [2]. Various truck-trailer lengths ranging from 10.1 to 25.25 m were compared. The aerodynamic impact is illustrated through vehicle stability and its effect on yaw. A specific aerodynamic influence of the semi-trailer rear side is highlighted by Kutuk et al. in their paper [3]. The authors analyzed the height and shape of the semi-trailer rear side using CFD simulations on a real dimensions model. They achieved a reduction in aerodynamic drag force of up to 9 %.

The significance of different aerodynamic devices on a semi-trailer truck is demonstrated by Pourasad et al. in their paper [4]. A large portion of the aerodynamic drag force (over 20 %) is generated by local drag behind the rear side of the trailer due to swirling airflow and low-pressure zones. The impact of aerodynamic devices at the rear side of the trailer is shown by Skrucany et al. in paper [5]. Except to describing the process of measuring aerodynamic drag force and the equipment used, the authors present experimental measurements on a scaled-down model in a wind tunnel and on a real vehicle on the road. The numerical research of the influence of crosswinds on the stability of a semi-trailer truck is depicted by Zhang et al. in their paper [6]. The large side surface area of the trailer is highly unfavorable in terms of generating side aerodynamic forces. Validation of the numerical calculations and CFD simulations was conducted through experimental measurements in a wind tunnel on a model six time scaled-down.

Another interesting 3D numerical study was conducted by Norouzi et al. in the paper [7]. The research focused on a medium-duty truck model with a semi-trailer. CFD simulation was performed using the Fluent software, employing the K-Omega turbulent flow model. The model was subjected to a constant airflow velocity of 30 m/s, and three zones were analyzed: above the cabin, below the cabin, and above the semi-trailer. In the research by Verzicco and colleagues in Ref. [8], the airflow around an ideal truck model is presented. The value of Reynolds number for the simulation was 10^5^, and large eddy simulation was used. Three different shapes of the rear side of the model were analyzed, and the results were validated with an experimental testing. Savkoor et al. present a dynamic analysis of an active aerodynamic device on the truck cabin in paper [9]. They used a standard NACA airfoil profile as a basis for designing and optimizing the active device. The purpose of the research and optimization process of the aerodynamic device is to reduce the vehicle's pitch.

The group of authors McCallen et al. in the paper [10] present the design approach for various aerodynamic devices for truck and semi-trailer models. They used the RANS model for CFD simulations. The validation of CFD simulation results was conducted through experimental testing on scaled-down models in a wind tunnel, as well as on real vehicles on the road. The authors particularly emphasize aerodynamic devices at the rear side of the semi-trailer in their research. The use of the K-Omega turbulent model for CFD simulation of airflow around and below a sports car model is demonstrated by authors Buljac et al. in the paper [11]. The research goal was to increase the lift force on the aerodynamic spoiler to enhance the load on the rear driving axle. Several configurations of the aerodynamic spoilers with airfoil profiles in base were employed. Galamboš et al. in the paper [12] present a 3D K-Epsilon turbulent flow model within the intake manifold of a spark-ignition engine, with Reynolds number values ranging between 7.7 × 10^4^ and 1.8 × 10^5^. Verification of simulation results was performed through experimental measurement of absolute pressure in the spark-ignition engine on a test bench. The blockage ratio is a key parameter in aerodynamic analyses, representing the ratio of the model's cross-sectional area to the cross-sectional area of the wind tunnel. A higher blockage ratio leads to artificially increased pressure and drag due to the constrained airflow around the model, potentially affecting the accuracy of the results. According to Hucho [13], maintaining lower blockage ratio values (<15 %) ensures that experimental results more accurately represent real-world conditions. According to Pope [14], high blockage ratio values (up to approximately 30 %) can be considered acceptable when testing non-lifted bodies, such as cylindrical shapes, or two-dimensional symmetrical wings. Additionally, the influence of wall and ceiling proximity in smaller wind tunnels designed for automotive testing has been analyzed through variable geometry configurations of the tunnel. This approach helps mitigate the effects of high blockage ratios and ensures reliable experimental outcomes even in constrained testing environments. A detailed optimization procedure of the geometric shape of emergency vehicle rotating lights is presented by Taherkhani in his PhD Thesis [15]. The optimization process combines the Design of Experiments method conducted through a series of CFD simulations and verification through experimental measurements on a real vehicle in a wind tunnel. A similar optimization approach, utilizing the Full Factorial Design and Response Surface Method within the Design of Experiments, is employed by Galamboš et al. in the paper [16]. The study focuses on an aerodynamic cabin spoiler of a scaled-down semi-trailer truck model. The optimization process was conducted through CFD simulations, and the verification was performed on a wooden scaled-down model in a wind tunnel.

Based on a review of relevant literature, the authors identify opportunities for further research in optimizing the geometric shape and position of an aerodynamic device positioned at the rear side of a semi-trailer. This study continues a broader, more comprehensive investigation that also underpins the findings presented in paper [16].

## 2 Testing object

This section of the paper combines: the presentation of the test object through a three-dimensional Computer Aided Design (3D CAD) model of a semi-trailer truck, the CFD model used for virtual experiments with detailed setup, and finally, the obtained results of the CFD simulations of the initial model.

### 2.1 CAD model

**Fig. 1:**
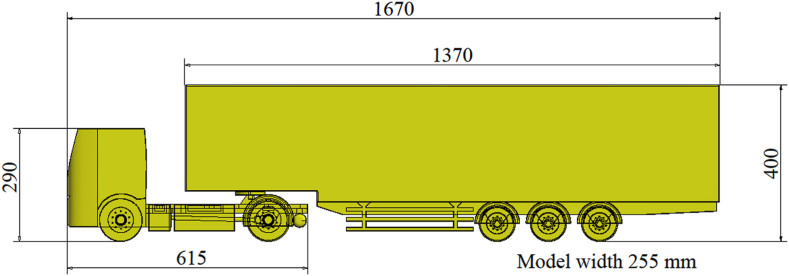
The CAD model of scaled semi-trailer truck.

The test object consists of a simplified 3D CAD model of a semi-trailer truck. The CAD model is based on the real model of a DAF truck and a Schwarzmüller semi-trailer. The CAD models are created in a 1:10 scale due to the experimental part of testing the model in a wind tunnel (which will be discussed in the upcoming sections of the paper). Fig. 1 shows the appearance of the semi-trailer truck CAD model with main dimensions in millimeters.

### 2.2 CFD model

The virtual testing of the aerodynamic characteristics of the model was performed using CD Adapco-Star CCM + software. The CAD models were imported into this software to conduct a detailed CFD analysis. A CAD model of a wind tunnel with a half-cylinder shape was created specifically for this purpose, with dimensions of 5 m in length and a radius of 0.6 m. While the chosen dimensions of this CAD model approximate those of the actual wind tunnel, they represent a simplified configuration. To minimize the impact of the stationary wind tunnel floor, the truck and semi-trailer model were elevated by 5 mm from the floor.

The boundary conditions within the CFD model of the wind tunnel are defined as Velocity Inlet for the incoming flow and a Pressure Outlet for the exhaust flow. Both the wind tunnel walls and the semi-trailer truck model are set as Wall boundaries.

The physical properties of the CFD simulation were chosen to significantly influence the accuracy of the results. A steady-state flow condition was assumed, with no thermal effects (Isothermal). The airflow velocities within the wind tunnel range from 60 to 90 km/h, resulting in Reynolds number values between 1.8 and 2.7 million, based on the CAD model length of approximately 1.7 m. These Reynolds number indicate that the flow is turbulent. The turbulent model employed is the Reynolds Averaged Navier Stokes (RANS) Realizable K-Epsilon model. The flow equations for pressure and velocity are solved using the Segregated approach with the Semi-Implicit Method for Pressure Linked Equations (SIMPLE) algorithm.

The All y + Wall treatment is applied in this simulation. This hybrid Wall treatment is employed when at least two mesh sizes are present. A coarse mesh is used for the wind tunnel model, while a finer mesh is applied to the semi-trailer truck model. The simulation is considered complete once the Residuals' root mean square value falls below 0.001. Throughout all simulations, the Drag Force and Drag Coefficient values are monitored. These values consistently are stabilize, remaining steady until the Residuals drop below the threshold of 0.001.

In addition to the physical settings of the CFD model, the proper selection and adjustment of the mesh are crucial to the simulation's accuracy and reliability. Following the recommendations in Ref. [17], a prismatic polyhedral volumetric mesh is adopted. This mesh type is suitable for models with complex geometries exposed to turbulent flow. While it is more computationally intensive than other mesh types, placing a greater demand on the computer, it ensures more stable and reliable results. The CFD model consists of a single region divided into two surfaces: the semi-trailer truck model as one surface and the wind tunnel model as the other. The base cell refers to the length of the edge of the cells that make up the mesh. In this research, two mesh sizes are used to optimize the simulation. One mesh size is applied to the wind tunnel model, which includes the inlet, outlet, and tunnel walls, while the other mesh size is used for the semi-trailer truck model.

The wind tunnel mesh size is adjusted directly through the base cell size. For the semi-trailer truck model, the mesh cell size is controlled using the target cell size and minimum cell size, both of which are defined as percentages relative to the base cell size of the wind tunnel model. According to recommendations from references [18,19], the target cell size is set at 10 %, while the minimum cell size is set to 5 % of the base cell size of the wind tunnel model.

**Fig. 2:**
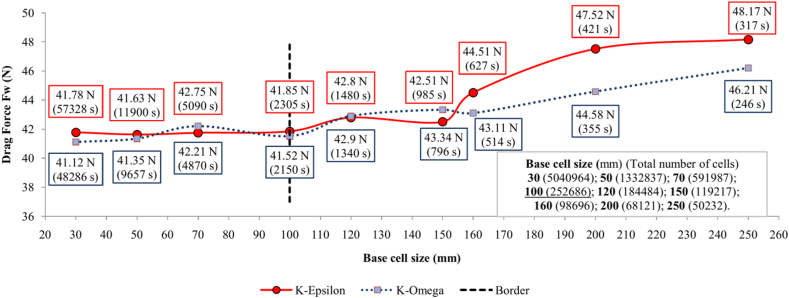
Mesh independence analysis.

The appropriate base cell size for the model was determined through a Mesh Independence Analysis (MIA). This analysis examines the dependency of the parameter of interest on the mesh size, along with other related parameters. For this study, MIA was conducted by monitoring changes in the Drag force values across different mesh base cell sizes. The base cell size is directly correlated to the number of cells in the model; as the base cell size decreases, the total number of cells increases, which can improve the accuracy of the results but also significantly slow down simulation execution. Therefore, it is necessary to find an optimal balance in base cell size where the results remain stable while not excessively prolonging the simulation time. In addition to the base cell size, the MIA also compared two types of turbulent flow models, K-Epsilon and K-Omega. The results of these comparisons are presented in the diagram of Fig. 2. The MIA was performed with base cell size ranging from 30 to 250 mm. Each simulation ran for 500 iterations at a single air velocity of 90 km/h. For every base cell size, two simulations were conducted: one using the K-Epsilon turbulent model and the other with the K-Omega model. In addition to Drag force as the primary output parameter, the total number of cells in the model and the simulation runtime (in seconds) were monitored. These results are presented in Fig. 2.

Since both K-Epsilon and K-Omega turbulent models were tested with the same base cell sizes, the total number of cells depends only on the base cell size, independent of the turbulent model. As shown in Fig. 2, increasing the base cell size up to 100 mm caused minimal variation in the Drag force value. However, above 100 mm, a notable increase in Drag force was observed. This trend appears consistently across both turbulent models. The range on the diagram in Fig. 2, with base cell sizes between 30 and 100 mm, displays very similar Drag force values.

The simulation runtime for a base cell size of 30 mm is nearly 25 times longer than that for a 100 mm base cell size, while producing a very similar Drag force result. However, when the base cell size exceeds 100 mm, the total number of cells and the simulation runtime are significantly reduced, but the Drag force result becomes notably variable. For base cell sizes above 100 mm, the K-Epsilon turbulent model shows a greater deviation in Drag force values compared to the K-Omega model. Additionally, the K-Omega model imposes a lower computational load, resulting in shorter simulation times. In some cases, simulations with the K-Epsilon model may reach stagnation, with residuals dropping below 10^−3^ after only 200 iterations. Conversely, simulations using the K-Omega model, sometimes require more than 500 iterations to stabilize.

Despite the advantages of the K-Omega turbulent model in terms of faster simulation and lower computational demands, the K-Epsilon model demonstrates superior result stability. Therefore, for all subsequent simulations in the research, the authors chose the K-Epsilon model with a base cell size of 100 mm.

### 2.3 Base model

**Fig. 3:**
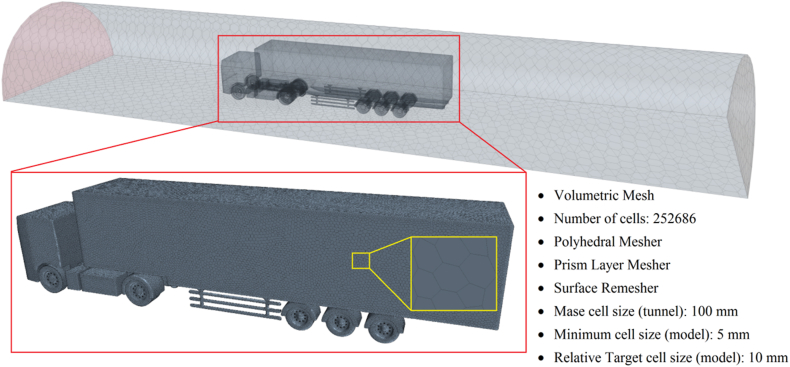
The CFD semi-trailer truck mesh model in the wind tunnel with main specifications.

**Fig. 4:**
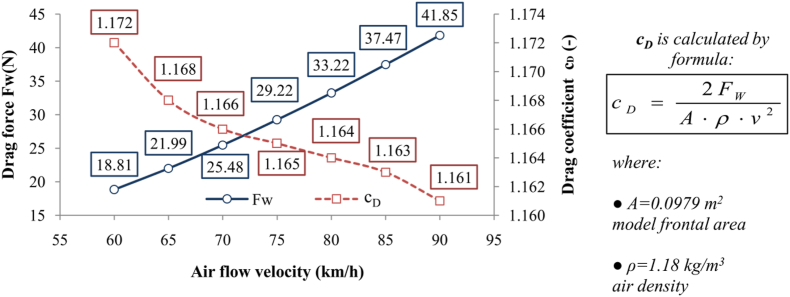
Drag force and drag coefficient of the base CFD model.

Fig. 3 illustrates the CFD mesh model of the semi-trailer truck within the wind tunnel. This model contains a total of 252,686 mesh cells and serves as the base model for the aerodynamic optimization procedure described subsequent sections. Seven simulations were conducted on the base model, each configured identically but tested at air velocities ranging from 60 to 90 km/h, in 5 km/h increments. The resulting values for the aerodynamic drag force and drag coefficient at these various velocities are displayed in the chart in Fig. 4. The observed trend of a slight decrease in the drag coefficient with increasing speed, as illustrated in Fig. 4, is characteristic of scaled models tested in controlled environments such as wind tunnels. Beyond the primary dependence of the drag coefficient on the model's geometry, numerous additional factors can influence its variation with speed, including turbulence models, boundary layer thickness, and transitional flow regimes [20].

## 3 The optimization procedure

This part of the paper demonstrates the adoption of the initial shape and position of the aerodynamic device at the rear side of the semi-trailer and the subsequent improvement process aimed to reduce the aerodynamic drag force of the entire semi-trailer truck model. The improvements to the initial shape and position of the aerodynamic device are performed using Design of Experiments (DoE). After adopting the basic shape and position of the aerodynamic device, the research proceeds with a Full Factorial Design involving four parameters, varying their physical values at three levels. This optimization step provides fundamental insights into the system's behavior and how the adopted parameters influence to the aerodynamic drag force.

The next step in the optimization process involves the Central Composite Design within the Response Surface Method. In this step, a smaller number of variables are taken into account but at a larger number of levels. This approach yields results closer to the optimal solution. The outcome of the Response Surface Method is an equation of the theoretical model, which, with the help of numerical quasi-Newton minimization, determines the optimal shape and position of the aerodynamic device based on the initial specified conditions.

The optimization process is carried out through a large number of virtual experiments using CFD simulations.

### 3.1 Initial adaptation

The existing CAD model of the semi-trailer truck is augmented with an aerodynamic device positioned at the rear side of the semi-trailer. The areas behind the rear side of the semi-trailer are characterized by the occurrence of swirling motion of air and low pressure, resulting in the creation of local drag force that constitute around 20 % of the total drag force of the vehicle. This information leads the authors to focus the continued research on the zone behind the semi-trailer model.

**Fig. 5:**
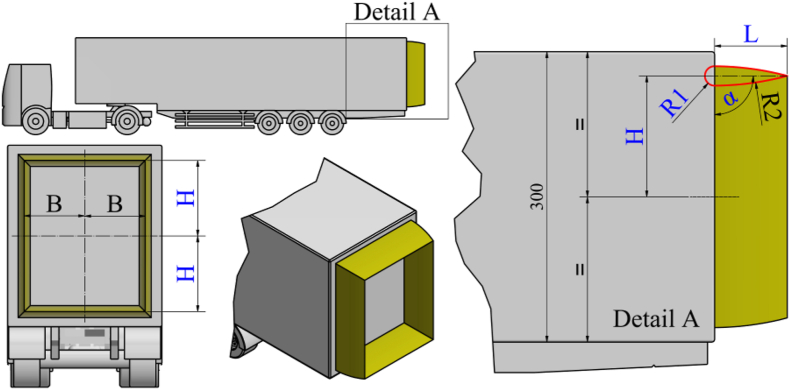
Initial shape and position with adopted parameters of the aerodynamic device on the rear side of the semi-trailer truck model.

For the purposes of the ongoing optimization procedure, the shape and position of the aerodynamic device are adopted, defined through four variable parameters and several initial adoptions. The four parameters are: α, H, L, and R1. Fig. 5 provides an illustration of the basic shape and position of the aerodynamic device at the rear side of the semi-trailer model in several views. The basic profile of the aerodynamic device is an airfoil symmetric biconvex about its mean chord line. The parameter α represents the angle of attack of the aerodynamic device profile and is located between the vertical rear side of the semi-trailer and the mean chord line of the device. The parameter H is the vertical distance between the mean chord line of the aerodynamic device profile and the midpoint of the height of the rear side of the semi-trailer. The parameter L represents the size of the protrusion of the aerodynamic device profile from the rear side of the semi-trailer along the mean chord line of the aerodynamic device profile. The parameter R1 is the radius of the leading edge of the aerodynamic device profile. In Fig. 5, the radius of curvature of the lower side of the profile R2 is also dimensioned. The value of this parameter is adopted as final and amounts to 0.3 m.

Defining the shape of the aerodynamic device profile involves its extrusion on all four sides of the surface of the rear side of the semi-trailer. Positioning the aerodynamic device at the rear side of the semi-trailer in a rear view is done by symmetrically separating from the center of the rear side of the semi-trailer through the variable H with respect to the vertical axis, and an additional parameter B along the horizontal axis. Each value of the parameter H corresponds to a unique change in the parameter B to achieve an equal distance of the aerodynamic device from all edges of the rear side of the semi-trailer.

### 3.2 Full Factorial Design

The parameters α, H, L, and R1 with adopted values at three levels are presented in Table 1.

**Table 1:** Full Factorial Design of Experiments - Parameter levels.

Level code	α (°)	H (mm)	L (mm)	R1 (mm)
−1	80	100	50	5
0	90	125	75	10
1	100	150	100	15

**Fig. 6:**
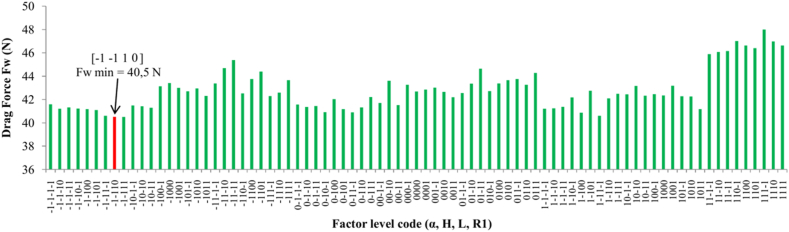
Results of full factorial design of experiments.

The four parameters with three levels of adopted values represent a total of 81 combinations of experiments. Fig. 6 shows the values of the aerodynamic drag force of the semi-trailer truck model for all experiment combinations. All CFD simulations in this phase of the optimization process are conducted for the highest considered airflow velocity of 90 km/h. Combination number 8 (−1, −1, 1, 0) yields the minimum value of aerodynamic drag force.

**Fig. 7:**
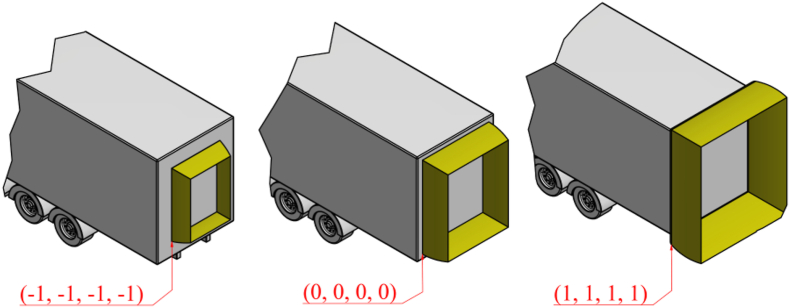
Shape and position of aerodynamic device in three typical configuration of parameters (α, H, L, R1) in Full Factorial Design.

Fig. 7 shows the design of the aerodynamic device for three typical configurations of parameters -1-1-1-1, 0000, and 1111.

**Fig. 8:**
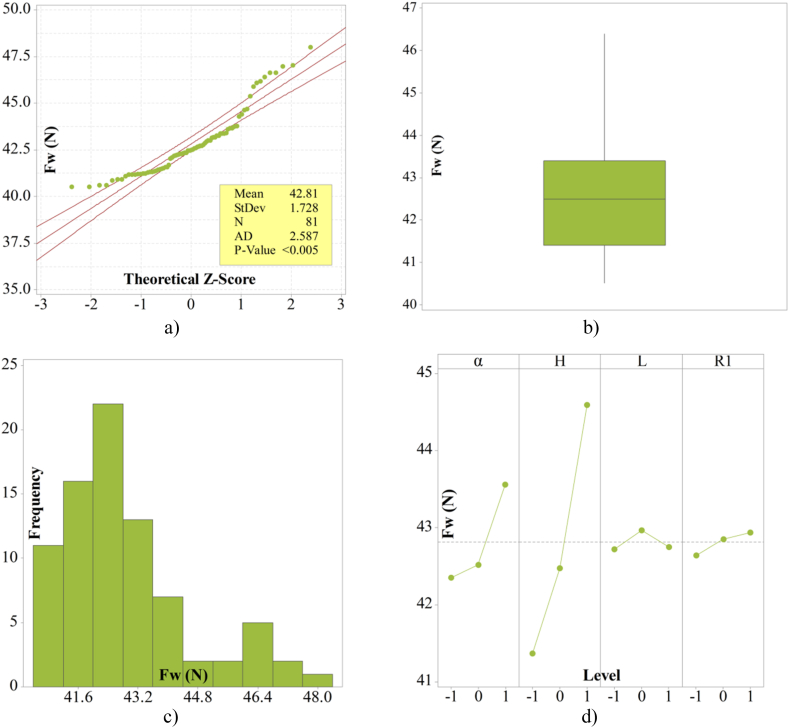
Descriptive statistic analysis of drag force in Full Factorial Design for the semi-trailer truck model with aerodynamic device a) Normal Probability Plot, 95 % Cl; b) Box Plot; c) Histogram; d) Main Effects Plot.

The diagram of results from Fig. 6 is accompanied by a statistical analysis of the results, which includes a Normal Probability Plot, Box Plot, Histogram, and Main Effects Plot. The statistical analysis of the results, conducted according to the recommendations in Refs. [21,22], is depicted through diagrams in Fig. 8.

Fig. 8a shows the Normal Probability Plot. The P-Value serves as a measure of the significance of the analyzed array of values. If its value is less than 5 %, the analyzed array of values is considered significant and justified from an analytical standpoint. The P-Value is less than 0.005 and indicating that the array of values for aerodynamic drag force is statistically significant. The Standard Deviation value is 1.728.

The Box Plot from Fig. 8b represents the zone of concentration of the results. The densest region of results is around the lower values of aerodynamic drag force, which is favorable for the nature of the research, as the optimization of the aerodynamic device aims to reduce the value of aerodynamic drag force.

Fig. 8c displays the Histogram, which shows the frequency of occurrence of the array of values from lowest to highest. The Histogram in Fig. 8c demonstrates the highest frequency of values in the array at lower values of aerodynamic drag force. Main Effect Plots illustrate graphically how each level of parameters influences the result. Fig. 8d shows how the adopted levels of variables affect the formation of aerodynamic drag force. Variable H has the most significant impact on the result of aerodynamic drag force, followed by variable α, while variables L and R1 have a lesser influence. This representation is crucial for a more detailed definition of the parameter levels in the next phase of the optimization process.

### 3.3 Response Surface Method

The next step of the optimization process involves increasing the number of levels of parameters defined in the Full Factorial Design. Based on the Mean Effect Plots from Fig. 8d, five new levels of parameters are defined, grouped around values that showed a tendency towards lower values of aerodynamic drag force. Parameter L from Fig. 8d has the smallest influence on aerodynamic drag force, so in this part of the optimization process, it is adopted as a final value corresponding to the −1 level from the Full Factorial Design.

For the continuation of the optimization process, the Central Composite Design (CCD) is used according to the recommendations from Refs. [23,24]. This method significantly reduces the number of conducted experiments. Three parameters at five levels represent 125 configurations if using the Full Factorial Design. With CCD, the number of combinations is much smaller, totaling only 20, of which the last six are the same combinations due to sharing the same central point by definition of CCD. Table 2 displays the three parameters α, H, and R1, and the newly adopted five levels of values.

**Table 2:** Central composite design - parameter levels.

Level code	α (°)	H (mm)	R1 (mm)
−2	70	70	3
−1	75	80	4
0	80	90	5
1	85	100	6
2	90	110	7

The obtained results of aerodynamic drag force and aerodynamic drag coefficient within the CCD are presented in Table 3.

**Fig. 9:**
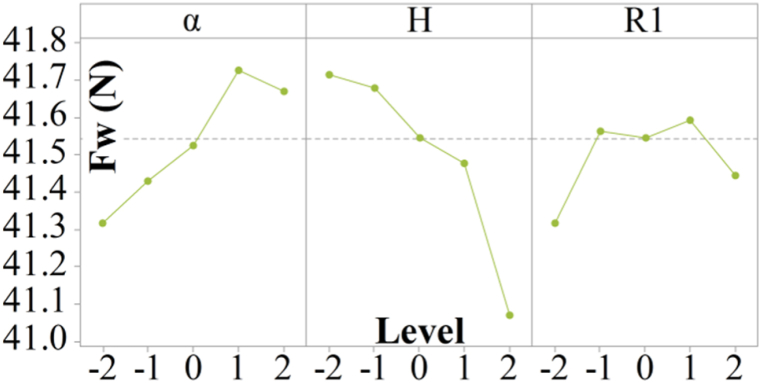
Main Effects Plot for five levels of parameters α, H and R1 at Central Composite Design.

Just like in the case of Full Factorial Design, the statistical analysis of the results from Table 3 was conducted similarly, with the authors opting to display only the Mean Effect Plots in this case. Fig. 9 shows the Mean Effect Plots for the results of the series of aerodynamic drag force values within the CCD.

**Table 3:** Results of central composite design.

Combination	Parameter level code			CFD simulation results	
	α	H	R1	Drag coefficient (−)	Drag force (N)
1	−1	−1	−1	1.160	41.89
2	1	−1	−1	1.149	41.51
3	−1	1	−1	1.147	41.41
4	1	1	−1	1.147	41.44
5	−1	−1	1	1.161	41.91
6	1	−1	1	1.147	41.40
7	−1	1	1	1.155	41.70
8	1	1	1	1.145	41.36
9	1	0	0	1.154	41.67
10	2	0	0	1.144	41.32
11	0	1	0	1.155	41.72
12	0	2	0	1.137	41.07
13	0	0	1	1.144	41.32
14	0	0	2	1.148	41.44
15	0	0	0	1.152	41.62
16	0	0	0	1.152	41.62
17	0	0	0	1.152	41.62
18	0	0	0	1.152	41.62
19	0	0	0	1.152	41.62
20	0	0	0	1.152	41.62

From Fig. 9, it can be observed how the adopted values of the parameter levels α, H, and R1 affect the aerodynamic drag force of the semi-trailer truck model. All three parameters, ranging from levels −2 to 2, show a slight influence on the change in aerodynamic drag force, below 1 N. This indicates that the level values are well adopted, and the aerodynamic drag force value approaches the optimal value with this type of aerodynamic device. Parameters α and R1 showed the lowest aerodynamic drag force values at levels −2, while parameter H showed it at level 2.

Using regression analysis of the results from Table 3, the coefficients of the Response Surface Method were obtained and are presented in Equation (1). This equation represents the theoretical model of the Response Surface Method and describes the system behavior with a precision of 89 % in an uncoded form.

(1) $${\text{Fw}}_{\text{th}}=41.718-0.030\cdot \alpha -0.184\cdot H+0.385\cdot R1--0.0210\cdot \alpha^{2}-0.0462\cdot H^{2}-0.0490\cdot R1^{2}++0.0725\cdot \alpha \cdot H-0.0601\cdot \alpha \cdot R1+0.0374\cdot H\cdot R1$$

**Fig. 10:**
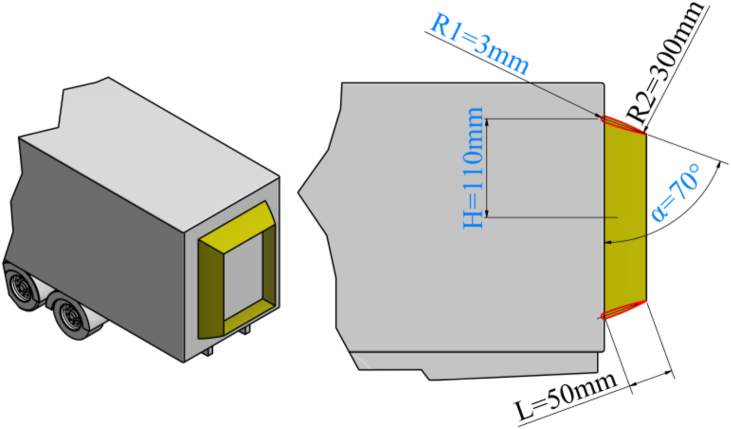
Aerodynamic device on the rear side of the semi-trailer in optimal position.

Using numerical quasi-Newton minimization with Equation (1), the minimum aerodynamic drag force value of 40.41 N is obtained. The parameter levels that lead to the lowest aerodynamic drag force value obtained by numerical quasi-Newton minimization align with the graphical representation in Fig. 9. Fig. 10 shows the optimal shape and position of the aerodynamic device at the rear side of the semi-trailer, with the indicated dimensions of the parameters.

## 4 Wind tunnel experiment

The experimental testing was performed in the "Miroslav Nenadović" wind tunnel at the Faculty of Mechanical Engineering, University of Belgrade, Serbia. This wind tunnel features a working section with 6 x 2.9 × 2.1 m and can achieve a maximum airflow velocity of approximately 420 km/h, driver by a four-blade propeller powered by a 200 kW electric motor. The tunnel operates as a closed-circuit system, providing circular airflow for consistent testing conditions.

**Fig. 11:**
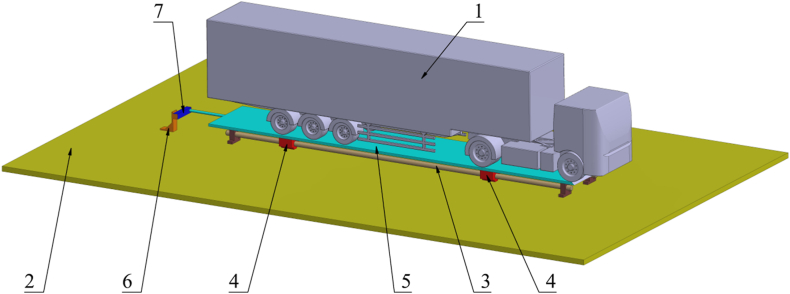
The measurement plant for measuring model drag force in the wind tunnel 1 - the semi-trailer truck model, 2 - the floor plate, 3 - sliding guides, 4 - sliders, 5 - the board, 6 - the force measuring cell holder, 7 - the force measuring cell.

The primary objective of this testing is to validate the results from the CFD simulations. To measure the aerodynamic drag force on the semi-trailer truck model within the wind tunnel, a custom measurement setup was developed, as depicted in Fig. 11. The aerodynamic drag force corresponds to the horizontal component of the resultant aerodynamic force acting on the model under airflow conditions, directed along its length of the vehicle. The truck and a semi-trailer model were constructed from wood based on the CAD model utilized in CFD simulations. The model's dimensions are 1670 x 255 × 400 mm, representing a 1:10 scale of the actual vehicle combination.

In addition to the semi-trailer truck model, labeled as item 1 in Fig. 11, the setup includes floor plate 2, which allows the entire measurement apparatus to be integrated into the wind tunnel floor, thereby exposing model 1 directly to the airflow. Attached to the floor plate 2 are sliding guides 3, which secure sliders 4 that can move along these guides. This sliding mechanism enables translational movement in the longitudinal direction of the model 1. To combine the truck model and semi-trailer model (which were fabricated separately), a board, labeled as item 5, is used. Model 1 is elevated by 5 mm above board 5 to reduce the influence of the stationary floor on the airflow around the model. The leading edge of board 5 is rounded to further streamline the flow. The gap between model 1 and board 5, along with the rounded shape of the board's leading edge, were selected based on a series of CFD simulations conducted during the initial testing phase. Model 1, along with board 5, has one degree of freedom of movement, which allows horizontal translational motion along the model's longitudinal axis. During experimental testing, the airflow impacts the model and pushes it backward. To measure the horizontal force, a rod is attached to the rear side of the board 5, transferring the horizontal force to the force-measuring cell 7. The cell is connected to the floor plate 2 via supports 6. The force-measuring cell 7 is a CZL623B-20 kg with a rated output of 2 ± 0.02 mV/V and a full-scale error of 0.03 %. The measurement signal from the force-measuring cell 7 is transmitted to the universal measuring amplifier HBM QuantumX MX840A. The data processing software used HBM catman Easy-AP ver. 3.5.1. This system allows for precise signal amplification and processing, ensuring the accuracy of the measured aerodynamic forces during the experimental testing.

**Fig. 12:**
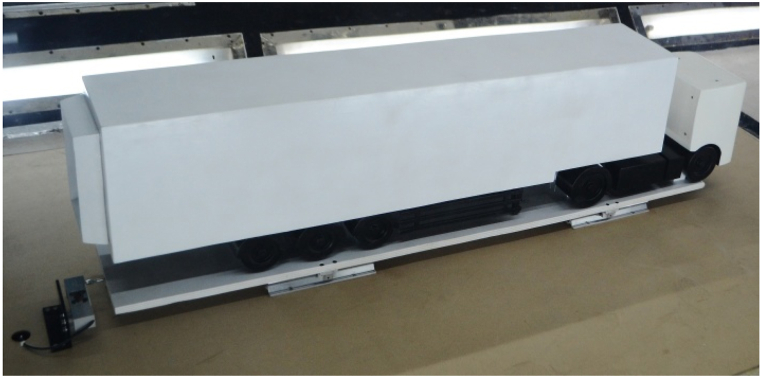
The semi-trailer truck model with aerodynamic device.

**Fig. 13:**
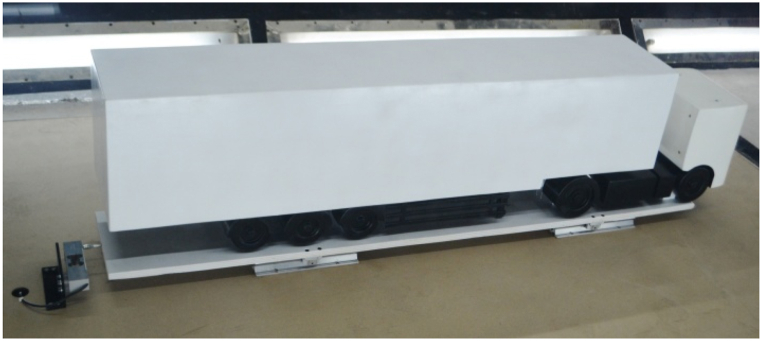
The semi-trailer truck model without aerodynamic device.

In the wind tunnel, aerodynamic drag force measurements were conducted for two different model configurations. The first configuration features the semi-trailer truck model with an aerodynamic device attached to the rear side of the semi-trailer, as shown in Fig. 12. The second configuration consists solely of the semi-trailer truck model without the aerodynamic device, shown in Fig. 13. For each configuration, aerodynamic drag force was measured at seven different air velocities, ranging from 60 to 90 km/h with increments of 5 km/h. Each measurement was repeated twice to ensure result consistency and repeatability. The data recording for each configuration and air velocity lasted approximately 20 s, allowing for accurate force measurements at each velocity.

## 5 Results

**Fig. 14:**
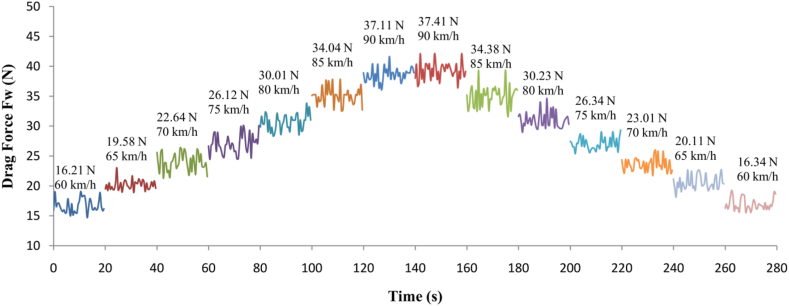
The measurement record of drag force for different air velocity regimes of the semi-trailer truck model with aerodynamic device.

The recorded data for the aerodynamic drag force, measured in the wind tunnel for the configuration with the aerodynamic device at the rear side of the semi-trailer, is shown in Fig. 14. The dataset includes aerodynamic drag force values across all considered air velocities, with repeated measurements for each. For clarity, above each graph corresponding to a specific air velocity, the average aerodynamic drag force is provided. In the paper, only the data for one configuration is presented, serving as a representative example of how the results from the experimental measurements of the aerodynamic drag force in the wind tunnel are processed and analyzed.

The verification of the results from the CFD simulations was carried out by experimentally measuring the aerodynamic drag force of the model in the wind tunnel. The CFD results were compared with the experimental data, and relative deviation between the two dataset was calculated using the following Equation (2). This equation quantifies the difference between the measured aerodynamic drag force (from the wind tunnel) and the CFD results, providing an indication of the accuracy of the simulations.

(2) $${\Delta FW}_{1,2}=\frac{{Fw}_{CFD}-{Fw}_{\mathrm{EXP}\;1,2\;}}{{Fw}_{\mathrm{EXP}\;1,2\;}}$$

In Equation (2), the component $Fw_{\mathrm{EXP}\;1,2}$ represents the value of relative deviation expressed in percentages. The component $Fw_{CFD}$ is the aerodynamic drag force value obtained from CFD simulation, while $Fw_{\mathrm{EXP}\;1,2}$ represents the aerodynamic drag force obtained from experimental measurements in the wind tunnel. In Equation (2), the indices 1,2 correspond to two experimental measurements, hence resulting in two values of relative deviation for each configuration and each air velocity.

Table 4 provides a comparison of the aerodynamic drag force for all considered model configurations and air velocities, with corresponding CFD simulation and experimental measurement results. The relative deviations provide insight into the accuracy of CFD model by quantifying the difference between the predicted and experimentally measured aerodynamic drag force values for each configuration and air velocity.

**Table 4:** Comparative representation of results.

*Configuration of the measuring facility*	*v(km/h)*	*F*_*W*__*CFD*_*(N)*	*c*_*D CFD*_*(-)*	*F*_*W EXP 1*_*(N)*	*c*_*D EXP 1*_*(-)*	*F*_*W EXP 2*_*(N)*	*c*_*D EXP 2*_*(-)*	*ΔF*_*W 1*_*(%)*	*ΔF*_*W 2*_*(%)*	*ΔF*_*W AVG.*_*(%)*
*The semi-trailer truck with aerodynamic device*	60	18.71	1.17	16.21	1.01	16.34	1.02	15.42	14.50	14.96
	65	21.47	1.14	19.58	1.04	20.11	1.07	9.65	6.76	8.21
	70	24.98	1.14	22.64	1.04	23.01	1.05	10.34	8.56	9.45
	75	28.51	1.14	26.12	1.04	26.34	1.05	9.15	8.24	8.69
	80	32.41	1.14	30.01	1.05	30.23	1.06	8.00	7.21	7.60
	85	36.37	1.13	34.04	1.06	34.38	1.07	6.84	5.79	6.32
	90	40.41	1.12	37.11	1.03	37.41	1.04	8.89	8.02	8.46
*The semi-trailer truck without aerodynamic device*	60	18.81	1.17	16.44	1.02	16.78	1.05	14.42	12.10	13.26
	65	21.99	1.17	20.01	1.06	20.23	1.07	9.90	8.70	9.30
	70	25.48	1.17	23.13	1.06	23.59	1.08	10.16	8.01	9.09
	75	29.22	1.17	26.87	1.07	27.09	1.08	8.75	7.86	8.30
	80	33.22	1.16	30.79	1.08	30.88	1.08	7.89	7.58	7.73
	85	37.47	1.16	34.95	1.08	35.17	1.09	7.21	6.54	6.87
	90	41.92	1.16	38.23	1.06	38.68	1.07	9.65	8.38	9.01

The average deviation between the aerodynamic drag force results obtained from CFD simulations and experimental measurements ranges from 6 to 15 %. In addition to the aerodynamic drag force, Table 4 also presents the drag coefficient c_D_. For the simulations, the drag coefficient is directly obtained from the software's output, while for the experimental measurements, it is computed using the equation for aerodynamic drag force. The introduction of the aerodynamic device at the rear side of the semi-trailer model results in an average improvement of approximately 3 % in its aerodynamic characteristics compared to the model without the aerodynamic device. This improvement is evidenced by a reduction in aerodynamic drag force. The relative deviation in the drag force values, whether from CFD or experimental measurements, remains similar for both models (with and without the aerodynamic device). The standard deviation of these results is, on average around 0.8, indicating relatively stable and consistent results across both methods of analysis.

The small deviation between the results obtained from CFD simulation and experimental measurements primarily reflects the accuracy and reliability of the CFD model. This success is largely attributed to the careful selection of the turbulent flow model (such as a K-Epsilon model) and the meticulous adjustment of the model mesh. The well-tuned CFD simulation effectively captured the dynamics of the real-world conditions simulated in the wind tunnel. Additionally, the experimental results demonstrate consistency and reliability, which can be attributed not only to the use of high-quality equipment but also to the thoughtful design and construction of the wooden semi-trailer truck model. The integrity of the entire testing setup, including the precision in both the measurement process and the modeling of the vehicle, further reinforces the accuracy of the results. This comprehensive approach ensures that the CFD model is a valid tool for simulating real-world aerodynamic behavior, validating its application for further optimization tasks.

**Fig. 15:**
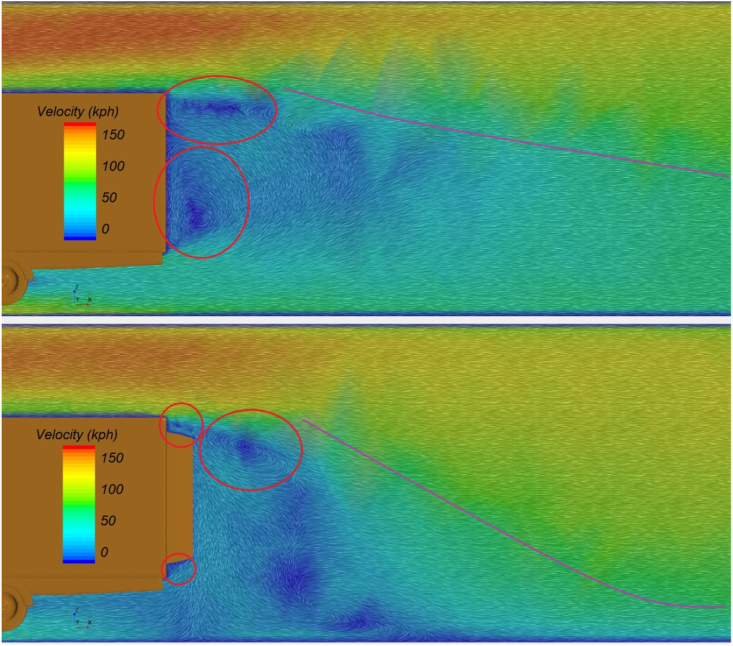
Vector velocity field around rear side of semi-trailer truck model without aerodynamic device (up) and with aerodynamic device (down).

Fig. 15 illustrates the airflow visualization around the rear end of a semi-trailer, both in the absence (upper image) and presence (lower image) of an aerodynamic device. The installation of the aerodynamic device notably alters the airflow patterns. The highlighted regions in the figure indicate areas where more intense vortex formation occurs. When the airflow encounters the rear end of the semi-trailer, it struggles to rapidly change direction, leading to the creation of a low-pressure zone and subsequent vortex formation. This vortex motion contributes to localized drag. The observed changes in airflow are primarily characterized by variations in the position and size of the vortices.

The highlighted regions in the upper part of Fig. 15 illustrate the presence of concentrated airflow vortices, which are centered around two large vortices. In contrast, the lower part of the figure demonstrates a more favorable airflow distribution behind the semi-trailer due to the aerodynamic device. These improvements are evident in the reduced vortex concentration, which corresponds to a lower localized drag force. The large vortices visible in the upper part of the figure are significantly diminished in the lower part, particularly the lower vortex. With the introduction of the aerodynamic device, this vortex splits into two smaller vortices, which can be observed on both the upper and lower sides of the device.

Another effect evident in Fig. 15 is the undisturbed diversion of airflow away from the rear edge of the semi-trailer. The dominance of the blue area in the upper part of the figure indicates a region of low airflow velocity, caused by the disruption of the primary airflow from both above and below the model. In contrast, the lower part of the figure shows a reduction in the blue area behind the semi-trailer, suggesting that the aerodynamic device minimizes airflow disturbance. This is further evidenced by the higher intensity of velocity vectors, which facilitate the re-establishment of steady airflow. To illustrate this phenomenon more clearly, the purple lines in Fig. 15 demarcate the boundary between the blue velocity zone and regions characterized by higher velocities.

## 6 Conclusion

In the context of this research, an optimization procedure was conducted to determine the shape and position of an aerodynamic device installed at the rear side of the semi-trailer truck model. An airfoil profile was selected as the basic shape of the aerodynamic device. The optimization procedure integrated Full Factorial Design within the Design of Experiments (DoE), Central Composite Design within the Response Surface Method (RSM), and numerical quasi-Newton minimization. The virtual aspect of the study was executed through CFD simulations, while the experimental phase was carried out in a wind tunnel. The experimental measurements in the wind tunnel served as a means of validating results obtained from simulations.

The aerodynamic device was characterized by four parameters: α, H, L, and R1, with each parameter varied across three levels. Statistical analysis of results obtained through Full Factorial Design revealed that the parameters α and H exert the most significant influence on the aerodynamic drag force. Conversely, the parameters L and R1 has a less pronounced effect on the aerodynamic drag force. This outcome is attributed to the geometric constraints imposed on the aerodynamic device, which limit the vehicle's overall dimensions to within 10 % of the original size.

Further refinement of the variable parameter levels was performed using the Central Composite Design, which provides a more precise understanding of each parameter's effect on the results. At five levels, all parameters produced a variation in the aerodynamic drag force on approximately 1 N. Utilizing the Response Surface Method, a quadratic equation for the theoretical model was derived. The final step in the optimization process involved numerical quasi-Newton minimization of the theoretical model equation. This process led to determination of optimal shape and position of the aerodynamic device, achieving a reduction in aerodynamic drag force by approximately 3 %, thus improving the aerodynamic characteristics of the model.

The optimization procedure discussed could yield even more substantial results if extended to include additional parameters and by targeting other regions of the semi-trailer truck model that may benefit for aerodynamic improvement.

Future research will focus on optimizing the aerodynamic characteristics of the area behind the truck cabin. This region, characterized by pronounced swirling motion and localized aerodynamic drag, presents a significant opportunity for improvement. By addressing this area, it is expected that further reductions in aerodynamic drag can be achieved, contributing to enhanced fuel efficiency and overall performance of the vehicle.

## CRediT authorship contribution statement

**Stjepan Galamboš:** Writing – original draft, Project administration, Methodology, Conceptualization. **Nebojša Nikolić:** Writing – review & editing, Supervision. **Goran Vorotović:** Resources, Investigation. **Boris Stojić:** Formal analysis. **Jovan Dorić:** Data curation. **Dalibor Feher:** Visualization.

## Declaration of competing interest

The authors declare the following financial interests/personal relationships which may be considered as potential competing interests: Nebojsa Nikolic reports financial support was provided by The Ministry of Science, Technological Development and Innovation of the Republic of Serbia. If there are other authors, they declare that they have no known competing financial interests or personal relationships that could have appeared to influence the work reported in this paper.
